# Detergents and alternatives in cryo-EM studies of membrane proteins

**DOI:** 10.3724/abbs.2022088

**Published:** 2022-07-20

**Authors:** Shuo Li

**Affiliations:** Department of Life Science National Natural Science Foundation of China Beijing 100085 China

**Keywords:** detergent, micelle, nanodiscs, cryo-EM, membrane protein

## Abstract

Structure determination of membrane proteins has been a long-standing challenge to understand the molecular basis of life processes. Detergents are widely used to study the structure and function of membrane proteins by various experimental methods, and the application of membrane mimetics is also a prevalent trend in the field of cryo-EM analysis. This review focuses on the widely-used detergents and corresponding properties and structures, and also discusses the growing interests in membrane mimetic systems used in cryo-EM studies, providing insights into the role of detergent alternatives in structure determination.

## Introduction

Integral membrane proteins play critical roles in many physiological processes including metabolism, signal transduction, and energy utilization [1], while structure determination of membrane proteins has been a long-standing challenge to understand the molecular basis of life processes. By the end of December 2021, there are totally 1401 unique membrane protein structures reported [2], and the coordinate files of transmembrane protein only account for 3% in all Protein Data Bank (PDB) entries [3].

Detergents are indispensable when working with integral membrane proteins. By nature of their amphiphilic character, detergents can partition into biological membranes, extract proteins, and maintain protein solubility in solution. The usage of detergents in membrane protein crystallization has been extensively reviewed over the last few decades [ 4– 8] , while single-particle cryo-EM has strongly affected the usage of detergents in recent years. The structure determination of membrane proteins has been specifically aided by the development of membrane mimetic systems. These advancements boost impressive growth in the number of single-particle cryo-EM membrane protein structures, and cryo-EM now rivals crystallography as the favored method for membrane protein structure determination [9].

This review first briefly discusses the basic properties of detergent and compare widely-used detergents between different experimental methods in recent years. This review then provides detailed insights into various effect of the detergent micelle on structure determination and corresponding structures, particularly in cryo-EM analysis. Here I further discuss the growing interests in membrane mimetic systems which provide a more native environment for integral membrane proteins, and focus particularly on the role of detergents alternative for structural and functional studies.

## Detergent of Choice

Detergents play an essential role in the extraction, purification, manipulation, and structure determination of membrane proteins. The amphiphilic characteristic of detergents allows them to interact with hydrophobic membrane proteins and to provide a native bilayer environment; it facilitates the extraction of proteins from membranes by disrupting the bilayer structure, then maintains the proteins in a soluble form [10]. The list of detergents used in membrane protein studies has expanded in last several decades, which can be grouped into seven broad categories, and more than 50 unique detergents are included. There is no standard setting for detergent usage, while the physical and chemical properties related to detergent function could guide the application on membrane proteins.

**Figure 1:**
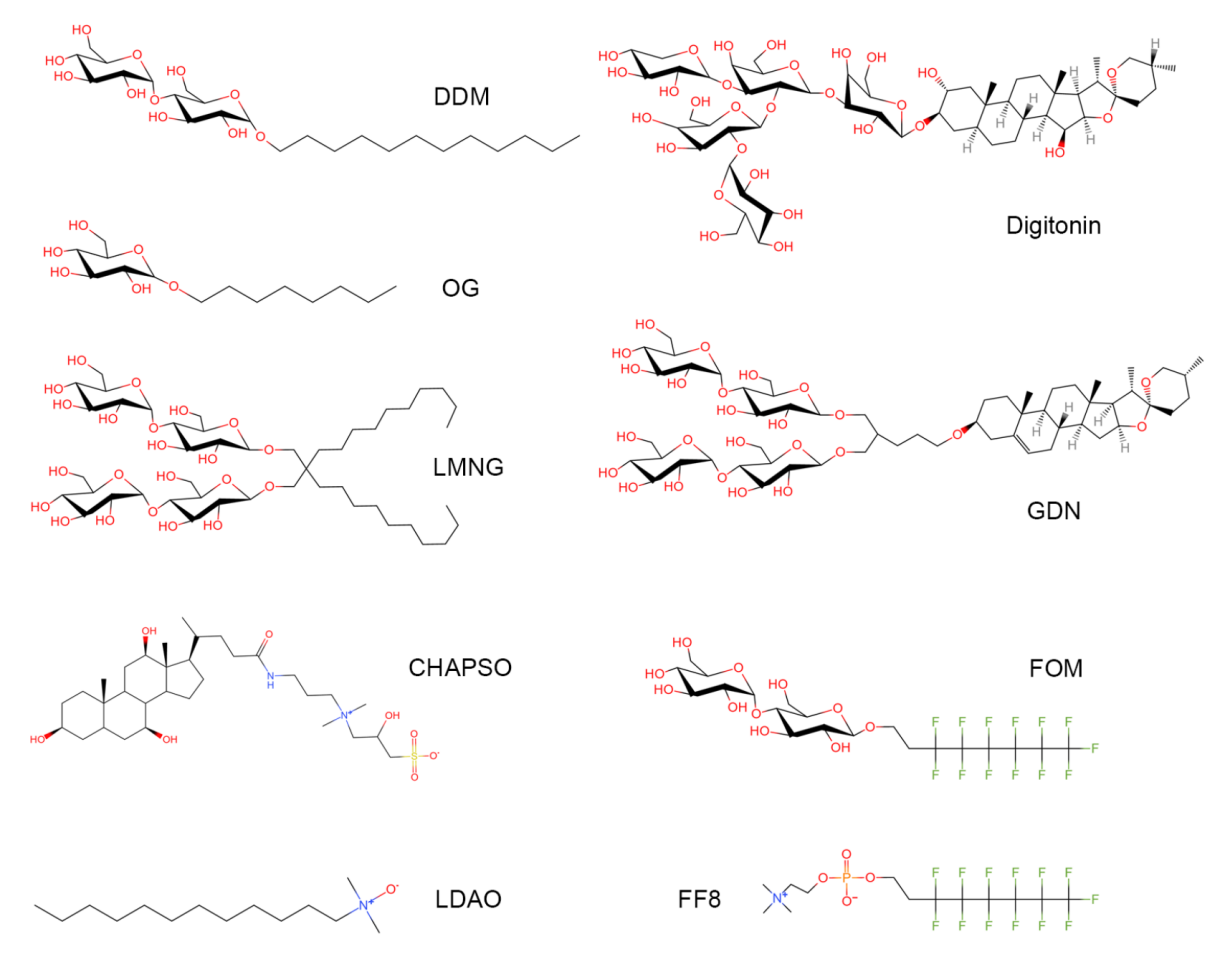
Figure 1 Representative detergent used in structure determination of membrane proteins Figure includes the detergents N-dodecyl β-D-maltoside (DDM), octyl glucoside (OG), lauryl maltose-neopentyl glycol (LMNG), lauryldimethylamine-N-oxide (LDAO), CHAPSO, digitonin, glyco-diosgenin (GDN), fluorinated octyl-maltoside (FOM) and fluorinated fos-choline-8 (FF8).

According to the property of hydrophilic head, detergents can be categorized into three groups: ionic, non-ionic, and zwitterionic detergents. Detergents with small, charged head groups and relatively short alkyl chains are generally much more denaturing to membrane protein structures [11]. Thus, ionic detergents, such as sodium dodecyl sulphate (SDS), are commonly used as a denaturant in studying membrane protein folding [ 12, 13] . Zwitterionic detergents are less chaotropic compared with the ionic ones. In fact, the zwitterionic lauryldimethylamine-N-oxide (LDAO) has been used for crystallization ( Figure 1) [ 14, 15] , although not very frequently, but often disrupt protein-protein interactions and cause denaturation of extracted membrane proteins [16]. Non-ionic detergents, including maltosides, glucosides, and polyoxyethylene glycols, tend only to disrupt the interactions between the protein and the lipids in the membrane. Most of the detergents useful in the purification and structural determination of membrane proteins are non-ionic detergents as well as the neopentyl glycol class detergents (i.e., LMNG) ( Figure 1) [ 4, 17, 18] , the latter typically possess lower critical micelle concentration (CMC) values than alkyl glycosides [ 19, 20] . According to the meta-analysis of unique membrane protein structures solved over the last decade, maltosides have been the detergent of choice for membrane protein solubilization in all kinds of experimental method, and glucosides are used less frequently [21].

Apart from these, different modes of solubilization selection appear when classifying structures by protein types and structural determination methods [22]. For example, cholesteryl hemisuccinate (CHS) is commonly used with combination of N-dodecyl β-D-maltoside (DDM) for eukaryotic membrane protein purification [ 18, 21, 23] , which functions as a membrane stabilizer [24]. The other detergent trend is an increasing application of digitonin and glyco-diosgenin (GDN), particularly in the area of single-particle cryo-EM studies ( Figure 1) [21]. First characterized in 2012, GDN is an amphiphile with a hydrophobic steroid-based group attached to a hydrophilic di-maltose head group [25]. As a synthetic drop-in substitute for digitonin [ 26, 27] , GDN has grown in popularity for reasons of cost and quality variability. A number of membrane protein structures have been determined by cryo-EM recently, where GDN was used for protein extraction or sample preparation and offered additional advantages in the studies [ 28– 32] .

Given that the optimal extraction conditions may not be the best conditions for further characterization [33], detergent exchange after membrane solubilization is widely applied before sample preparation across all experimental methods [ 7, 8, 34] . For example, maltosides with and without the combination of CHS represent the solubilization detergent compositions of choice for ~60% of the structures solved in the last decade, with a reduction to ~38.3% at the stage of structure determination [21]. High CMC detergents are more favored for crystallization because they benefits crystal contact formation, while detergents with low CMC are preferred by cryo-EM and I will discuss in detail in next section.

In addition, some detergents are often added to protein to improve grid preparation in cryo-EM samples. For example, zwitterionic detergent CHAPSO significantly broadens the particle orientation distributions for a number of bacterial transcription complexes ( Figure 1) [ 35, 36] , which alters the air-water interface causing several issues like protein denaturation, complex dissociation and preferred orientation [37]. Fluorinated detergents, fluorinated fos-choline-8 and fluorinated octyl-maltoside (FOM), have been reported to improve the distribution of membrane protein molecules in ice ( Figure 1) [ 38– 41] .

## Effect of the Micelle in Cryo-EM

Micellization is a critical phenomenon when considering detergent application. The concentration of detergent above which micelles form and all additional detergents added to the system go to micelles is described as CMC. Detergents with lower CMC are desirable in cryo-EM studies, such as GDN and LMNG, for which can minimize the detergent concentration while keep the protein soluble. Here I consider the effect of micelle on structure determination, particularly in the field of single particle cryo-EM.

In most cases, detergent levels in membrane protein samples are typically kept above the CMC to allow the hydrophobic parts of the protein incorporated into micelles [6]. But most commonly used detergents produce significant background in electron microscope imaging, caused by empty detergent micelles, even at a concentration below nominal CMC [42]. The free micelles are difficult to distinguish from membrane proteins in cryo-EM images, especially for small size proteins [43], which cause interference in particle picking, classification, and alignment. Taking into consideration that the optimal concentrations of total detergents for cryo-EM grids is around 0.2%–0.4%, several methods have been explored to minimize detergent concentration and avoid extra micelles in membrane protein samples. For example, GraDeR use glycerol gradient centrifugation to mildly remove free detergent monomers and micelles from LMNG, resulting in monodisperse and stable protein-detergent complex [44]. In some cases, the concentration of detergents is gradually reduced at each step of membrane solubilization, affinity chromatography and gel filtration, to grantee the final content of detergent in the sample for grid preparation [ 45, 46] .

On the other hand, a clearly delineated micelle may aid in the single particle classification and alignment. Micelle size varies with different detergents and concentrations, even not all micelles appear as spherical. For example, LMNG appears as worm-like filament in negative-stain images [42] and artefacts are apparent even at the concentration above 0.1% [22], which may interfere with protein particles. LDAO appears to have very small and weakly scattering micelles [47]. Digitonin has been successfully used for cryo-EM structure determination partially due to its well-defined micelle in images [ 48, 49] , which may be related to the composition of the hydrophilic headgroup compared to its hydrophobic tail. The addition of CHS to maltosides can enlarge the micelle and increase thickness of the hydrophilic layer [50], which provides a more native stabilizing environment and facilitate the identification of embedded small membrane protein. There are cryo-EM structures illustrating the molecular mechanism of small transmembrane regions in the condition of DDM with CHS [ 51– 53] .

## Novel Membrane Mimetic Systems Used in Cryo-EM

**Figure 2:**
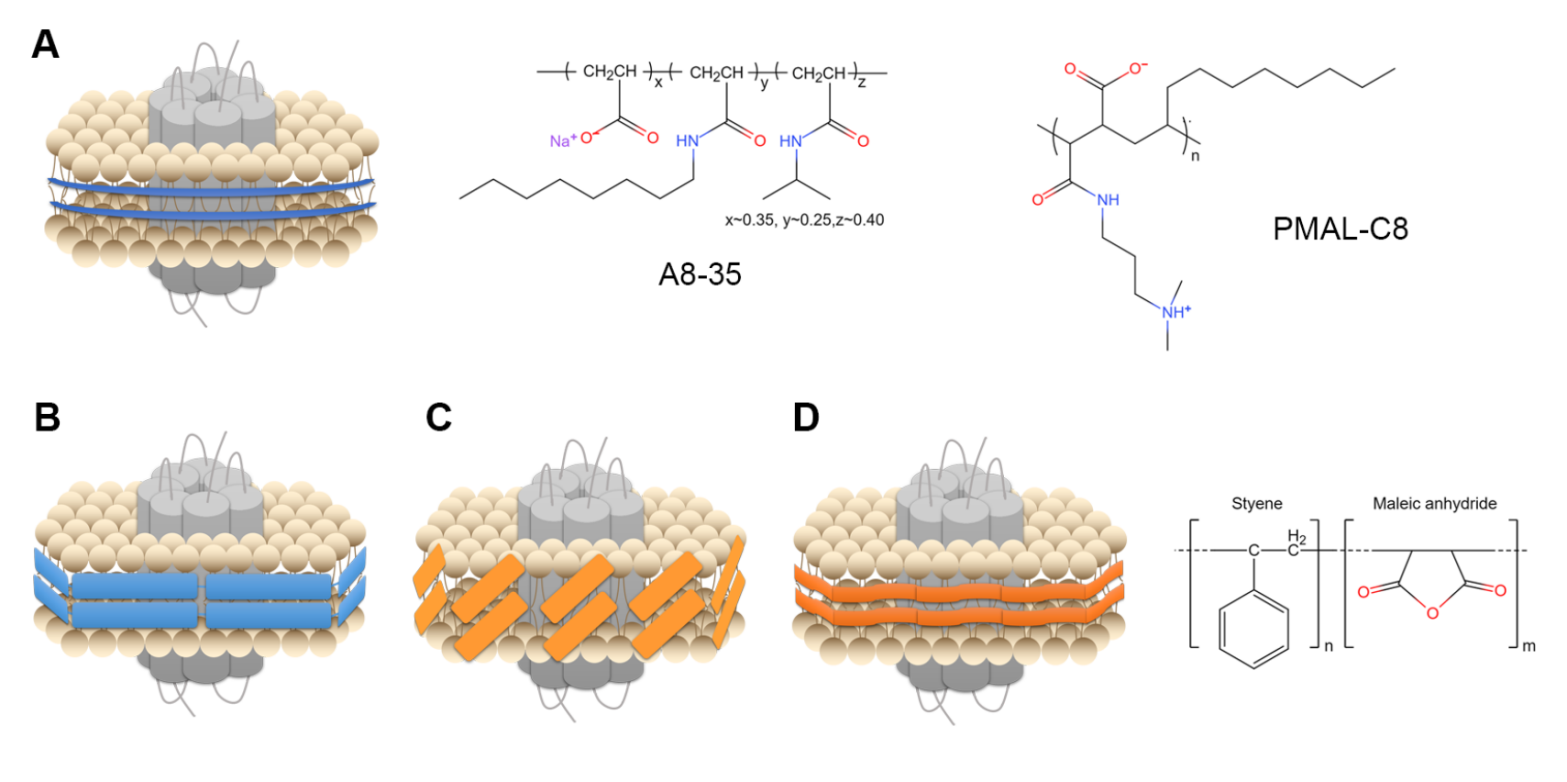
Figure 2 Schematic diagram of membrane mimetic systems Side view of (A) amphipols, (B) MSP nanodiscs, (C) saposin-lipoprotein, and (D) styrene maleic acid (SMA). The units of A8-35, PMAL-C8, and SMA-copolymer are shown, respectively.

The application of detergents in cryo-EM studies often causes complications such as increasing thickness of vitrified ice, reducing contrast, making background noise and particle distribution problems. To date, several novel membrane mimetic systems without the presence of detergents have been introduced to study integral membrane proteins ( Figure 2), including amphipols [ 54, 55] , nanodiscs [56], saposin-lipoprotein [57], and styrene maleic acid (SMA) [58]. These membrane mimetic systems are able to stabilize proteins in aqueous solution under their native states, facilitating the study of biological and biophysical properties of membrane proteins.

In the past few years, the most extensively used detergents alternatives are nanodiscs and amphipols [21]. Amphipols are a new class of surfactants which possess strongly hydrophilic backbones grafted with hydrophobic chains ( Figure 2A). The high affinity for the hydrophobic transmembrane domain makes amphipols advantageous for single particle cryo-EM analysis. The first cryo-EM structures of an integral membrane protein, TRPV1, were solved using amphipols as the stabilizing medium, and captured in two different conformational states [ 59, 60] . The most commonly used amphipols are A8-35 and PMAL-C8 ( Figure 2A) [ 61– 64] .

Nanodiscs consist of discoidal phospholipid bilayers stabilized by two belt-like membrane scaffold proteins (MSPs) derived from apolipoprotein A-1 ( Figure 2B) [ 65, 66] . For membrane proteins, nanodiscs can help to avoid aggregation and provide a more native environment by reconstituted lipid bilayers [67]. The distinct shape of MSP may also helpful for image alignment in data processing. The cryo-EM structures of TRPV1 with agonist bound were better solved at resolution of 2.9 Å using lipid nanodiscs as the stabilizing medium [68].

Reconstitution of most membrane mimetic systems requires solubilization and purification of membrane proteins in detergents at first. By contrast, SMA copolymers can directly insert into the bilayer and extract integral membrane proteins within natural lipids ( Figure 2D) [ 69, 70] , which potentially maintains the native environment and biological features. This method has been used in cosmetics and pharmaceutical research [71], but still rarely successfully applied in cryo-EM studies to obtain high-resolution structure of mammalian membrane proteins [72].

**Figure 3:**
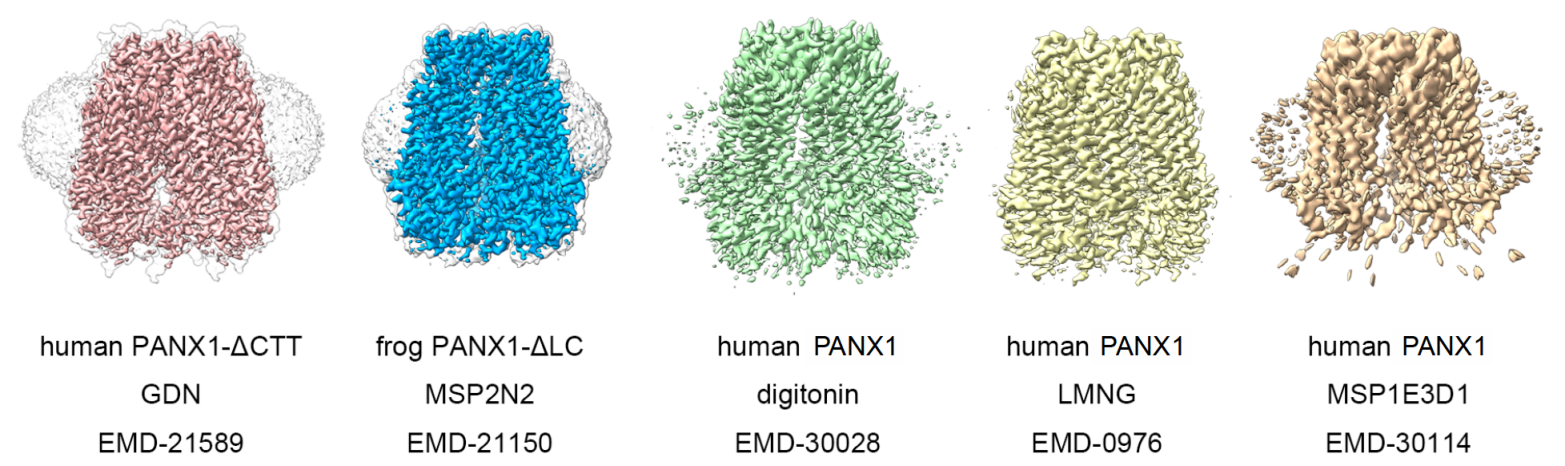
Figure 3 PANX1 structures in different membrane systems The figure demonstrates a selection of PANX1 structures recently determined in detergents or nanodiscs [ 75– 79] . The cryo-EM maps are presented in their recommended contour level and viewed parallel to the membrane. The unsharpened maps are shown as transparent envelope if available.

All the solubilization systems mentioned above are proved to be reasonable and practical, which can be successfully applied to structure determination of membrane proteins. Here I introduce an example of pannexin1, illustrating one membrane protein structure in parallel, to confirm the consistency of different detergents and membrane mimetic systems. Pannexin1 (PANX1) is an ATP-permeable channel with crucial roles in physiological functions such as regulation of blood pressure [73] and apoptotic cell clearance [74]. However, little is known about the molecular basis and inhibition mechanism of PANX1. Recently, there are a series of PNAX1 cryo-EM structures reported with a resolution above 4 Å [ 75– 79] , solved in different membrane systems but revealing the same gating mechanism ( Figure 3). In one of the outstanding research projects, the biochemical properties of purified human PANX1 protein were analyzed in both detergent and SMAs at first. The authors finally decided to focus on PANX1 in GDN, yielding cryo-EM maps at substantially higher resolution than those in SMA, while the heptameric stoichiometry of PANX1 was also confirmed by the SMA-extracted structure [75]. Another research presents the cryo-EM structure of a frog PANX1 channel in nanodisc MSP2N2 [76]. The other three human PANX1 structures are determined in digitonin [77], LMNG [78], and nanodisc MSP1E3D1 [79], respectively. The unsharpened cryo-EM maps show a strong-scattering detergent micelle or nanodiscs, and the features of protein regions are better depicted in final maps ( Figure 3).

## Perspectives

With technical development and facility renewal, single-particle cryo-EM has strongly affected the usage of detergents and membrane mimetics, resulting in a spurt of membrane protein structure determined. Conventionally used detergents, despite its deficiencies, is undeniably still a major force for the purpose of high-resolution structure determination. Therefore, the choice of detergents and alternatives should be considered comprehensively according to the experimental methods, protein type, and research target. This review also summarized representative conditions used for membrane protein structure determination by cryo-EM mainly mentioned in this review ( Table 1), which include protein name and family, protein size and structure resolution. Nevertheless, more developing approaches of biomembrane reconstitution, such as proteoliposomes [ 80, 81] , will apply to membrane protein studies and facilitate the cryo-EM structure determination in lipid bilayer environment.

**Table:** **Table 1** Recent unique membrane protein structures determined by cryo-EM in different systems

Name	Family	MW (kDa)	Resolution (Å)	Membrane mimetic systems	Additive	PDB code	Reference
Activated glucagon-like peptide-1 receptor in complex with G protein	GPCR: Class B1	162	4.10	0.01% LMNG, 0.01% GDN, 0.00192% POPG, 0.0012% cholesterol	/	5VAI	[29]
Alternative complex III (ACIII)	Electron transport chain complexes: complex III (Cytochrome bc1)	301	3.40	1% SMA 3000HNA	/	6BTM	[72]
Calcitonin receptor-hetero-trimeric Gs protein complex	GPCR: Class B1	164	4.10	0.01% LMNG, 0.006% CHS	/	5UZ7	[82]
Cyclic-nucleotide-gated channel	Channels: Potassium,sodium & proton ion-selective	213	4.20	LMNG, CHS	/	5V4S	[83]
Cystic fibrosis transmembrane conductance regulator (CFTR)	ABC transporters	170	3.73	0.06% Digitonin	3 mM FFC8	5UAR	[48]
Electron transport chain (ETC) super complexIII2IV2	Electron transport chain supercomplexes (Respirasome)	936	3.35	0.05% GDN	/	6HU9	[32]
HCN4	Channels: Potassium,sodium & proton ion-selective	393	3.60	A8-35	/	7NMN	[84]
Mitochondrial ATP synthase	F-type ATPase	385	3.60	0.02% GDN	/	6B2Z	[28]
Mitochondrial calciumuniporter	Channels: Calciumion-selective	212	3.70	PMAL-C8	/	6DT0	[82]
MRP1 multidrug resistance protein 1	ABC transporters	160	3.49	0.06% Digitionin	3 mM FFC8	5UJ9	[39]
MscS	Mechanosensitivechannel	239	3.10	MSP1 E3D1	0.01% FOM	6PWN	[40]
OSCA 1.1	Mechanosensitivechannel	175	3.50	0.1% Digitonin	/	6JPF	[85]
Pannexin 1 (Panx1) ATPrelease channel	Channels: other ionchannels	346	3.10	0.01% LMNG	/	6LTO	[78]
Pannexin 1 (Panx1) ATPrelease channel	Channels: other ionchannels	337	3.20	0.1% Digitonin	/	6M02	[77]
Pannexin 1 (Panx1) ATPrelease channel	Channels: other ionchannels	348	4.10	MSP1 E3D1	/	6M66	[79]
Pannexin 1 (Panx1) ATPrelease channel	Channels: other ionchannels	276	3.02	MSP2N2	/	6VD7	[76]
Pannexin 1 (Panx1) ATPrelease channel	Channels: other ionchannels	323	2.97	0.01% GDN	/	6WBG	[75]
Patched1 (Ptch1) of the Hedgehog (Hh) signaling pathway	Multi-drug efflux transporters	147	3.60	PMAL-C8	/	6MG8	[63]
Patched1 (Ptch1) of the Hedgehog (Hh) signaling pathway	Multi-drug efflux transporters	163	3.50	0.06% Digitonin	/	6OEU	[49]
P-Glycoprotein multi-drug transporter (ABCB1)	ABC transporters	144	3.40	0.05% DDM, 0.005% CHS	/	6C0V	[51]
Piezo2	Mechanosensitivechannel	981	3.80	0.02% GDN	0.65 mM FFC8	6KG7	[41]
Respiratory Complex I	Electron transport chain complexes: complex I	1066	3.30	0.5% DDM	/	6G2J	[86]
Serotonin transporter in complex with paroxetine	Neurotransmitter sodium symporter (NSS) family	87	4.20	1 mM DDM, 0.2 mM CHS	/	6DZV	[53]
SLC7A5 L-type amino acid transporter LAT1 in com-plex with 4F2hc (SLC3A2)	SLC transportersuperfamily	131	3.50	0.08% Digitonin	/	6IRT	[45]
STING (aka TMEM173, MITA, ERIS, or MPHYS)	Host-defense proteins	86	4.10	0.02% DDM, 0.004% CHS	/	6NT5	[52]
TMEM16	TMEM 16	167	3.60	0.03% DDM	/	6QM5	[87]
TOM complex	Mitochondrial outer membrane beta barrel proteins	192	3.06	0.03%DDM, 0.006%CHS	3 mM FFC8	6UCU	[88]
TRPA1	TRP channel	512	2.60	0.005% LMNG	/	6V9V	[89]
TRPC4	TRP channel	427	3.60	A8-35	/	6G1K	[90]
Two-pore channel TPC1	Channels: Potassium,sodium & proton ion-selective	192	3.40	0.06% GDN	/	6C96	[31]
γ-secretase	Intramembrane proteases	178	3.40	A8-35	/	5A63	[62]
γ-secretase nicastrinextracellular domain	Intramembrane proteases	178	4.50	A8-35	/	4UPC	[61]
μ-opioid receptor-Gi protein complex with scFv-16	GPCR: Class A	154	3.50	0.00075% LMNG, 0.00025% GDN, 0.0001% CHS	/	6DDE	[30]
